# IL-1β–Induced Protection of Keratinocytes against *Staphylococcus aureus*-Secreted Proteases Is Mediated by Human β-Defensin 2

**DOI:** 10.1016/j.jid.2016.08.025

**Published:** 2017-01

**Authors:** Bingjie Wang, Brian J. McHugh, Ayub Qureshi, Dominic J. Campopiano, David J. Clarke, J. Ross Fitzgerald, Julia R. Dorin, Richard Weller, Donald J. Davidson

**Affiliations:** 1 MRC Centre for Inflammation Research, Queen’s Medical Research Institute, The University of Edinburgh, Edinburgh, UK; 2 School of Chemistry, University of Edinburgh, Edinburgh, UK; 3 The Roslin Institute and Edinburgh Infectious Diseases, University of Edinburgh, Easter Bush, Midlothian, UK

**Keywords:** AD, atopic dermatitis, hBD, human β-defensin, HDP, host defense peptide, HPEK, human primary epidermal keratinocyte, LPS, lipopolysaccharide, LTA, lipoteichoic acid, OE, overexpressing, shRNA, small hairpin RNA, Ssp, staphylococcus aureus serine protease, TJ, tight junction, VO, vector only

## Abstract

Atopic dermatitis (AD) is a common chronic inflammatory skin disease that results in significant morbidity. A hallmark of AD is disruption of the critical barrier function of upper epidermal layers, causatively linked to environmental stimuli, genetics, and infection, and a critical current target for the development of new therapeutic and prophylactic interventions. *Staphylococcus aureus* is an AD-associated pathogen producing virulence factors that induce skin barrier disruption in vivo and contribute to AD pathogenesis. We show, using immortalized and primary keratinocytes, that *S. aureus* protease SspA/V8 is the dominant secreted factor (in laboratory and AD clinical strains of *S. aureus*) inducing barrier integrity impairment and tight junction damage. V8-induced integrity damage was inhibited by an IL-1β–mediated mechanism, independent of effects on claudin-1. Induction of keratinocyte expression of the antimicrobial/host defense peptide human β-defensin 2 (hBD2) was found to be the mechanism underpinning this protective effect. Endogenous hBD2 expression was required and sufficient for protection against V8 protease-mediated integrity damage, and exogenous application of hBD2 was protective. This modulatory property of hBD2, unrelated to antibacterial effects, gives new significance to the defective induction of hBD2 in the barrier-defective skin lesions of AD and indicates therapeutic potential.

## Introduction

Atopic dermatitis (AD) is a common relapsing inflammatory skin disease affecting 15–20% of children (Asher et al., 2006) and 2–10% of adults (Bieber, 2008) worldwide (<10.7% and 10.2%, respectively, in the United States) (Shaw et al., 2011, Silverberg and Hanifin, 2013) with significant morbidity. The disease is characterized by chronic, inflamed, itchy lesions, typically on the face, scalp, and limbs. A key hallmark of AD is disruption of upper epidermal layer barrier function, causatively linked to environmental stimuli, genetics (e.g., *FLG* mutations), and infections (Bieber, 2008).

AD lesions are particularly susceptible to infection with *Staphylococcus aureus* (Bieber, 2008, Hata and Gallo, 2008). *S. aureus* is found on the skin of less than 30% of healthy individuals, mainly in intertriginous areas (Park et al., 2013). However, 75–100% of AD patients have *S. aureus* on their lesional skin, and 30–100% of AD patients have *S. aureus* on their nonlesional skin (Breuer et al., 2002, Gong et al., 2006, Park et al., 2013). *S. aureus* secrete a range of virulence factors, including extracellular toxins and proteases, that contribute to disease pathogenesis (Nakamura et al., 2013). Serine protease A (SspA), also known as V8 protease, is a well-characterized extracellular protease widely expressed among different *S. aureus* strains (Drapeau et al., 1972, Kolar et al., 2013). The pathogenic effect of V8 protease has been shown in vivo, damaging murine skin integrity via effects on stratum corneum (Hirasawa et al., 2010, Ohnemus et al., 2008). Although the targets for V8-mediated damage remain unclear, *S. aureus* extracellular proteases can cleave tight junction (TJ) proteins, including claudins, occludin and ZO-1, and desmogleins (Hirasawa et al., 2010, Ohnemus et al., 2008), aggravating skin barrier dysfunction. The capacity of proteases to induce barrier dysfunction is proposed as a key driving force in the initiation and exacerbation of AD (Takai and Ikeda, 2011). Thus, understanding the host factors that maintain barrier function is a priority in developing therapeutic approaches.

AD is also characterized by the altered expression of antimicrobial/host defense peptides (HDPs), including members of the β-defensin family. β-Defensins human β-defensin (hBD) 2 and hBD3 are induced by skin wounding and inflammatory stimuli and are highly expressed in inflamed psoriatic skin lesions. In contrast, β-defensins are present at significantly lower levels in the inflamed skin lesions from individuals with AD (Ong et al., 2002). Although some β-defensin induction is observed in AD lesions compared with nonlesional skin (Harder et al., 2010), impaired β-defensin induction and/or release in AD is proposed to result from the effects of T helper type 2 cytokines and to be insufficient to protect against infection. The expression of HDPs in psoriasis and AD correlates with susceptibility to infection (Breuer et al., 2002, Schauber and Gallo, 2009), suggesting a key microbicidal role. However, these HDPs also have a wide range of modulatory activities, raising the possibility of additional roles in skin host defense (Cox et al., 2014).

Here we show that V8 is the dominant factor in *S. aureus*-mediated keratinocyte barrier integrity damage, that IL-1β can induce keratinocyte production of factors with potent protective activity, and that hBD2 is required and sufficient for this protection. These data show a protective function for the antimicrobial HDP hBD2, informing potential therapeutic approaches to prevent *S. aureus*-mediated aggravation of skin barrier dysfunction in AD.

## Results

### *S. aureus*-secreted proteases damage keratinocyte barrier integrity

**Figure 1:**
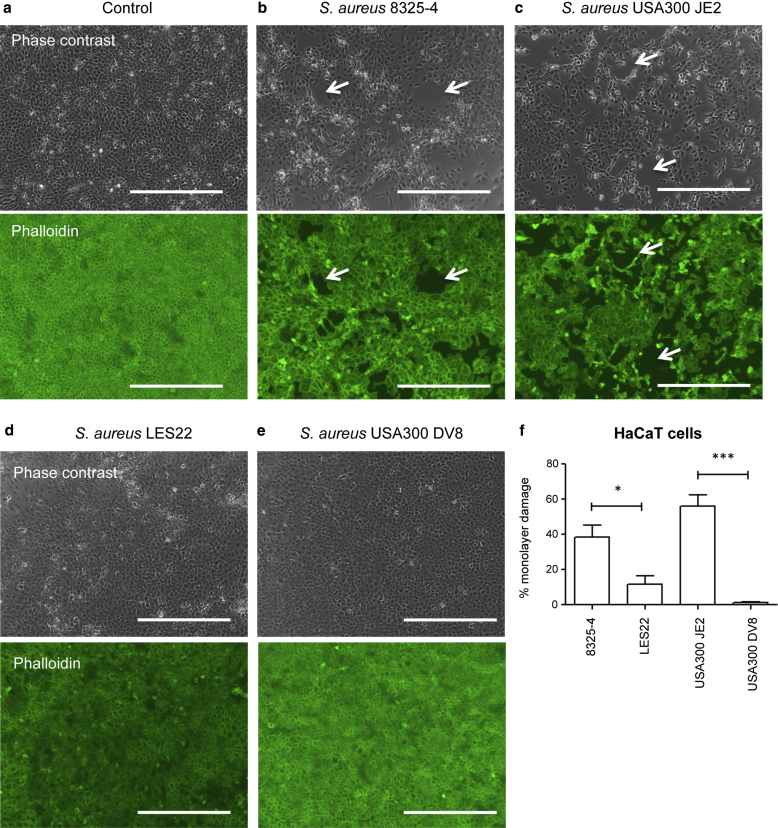
***Staphylococcus aureus*-secreted proteases damage keratinocyte barrier integrity.** HaCaT keratinocyte cultures treated with either (**a**) media only or (**b–e**) filtered *S. aureus* supernatant for 24 hours. Cells are shown by phase contrast microscopy (top row) or Alexa488 phalloidin stained (bottom row). Scale bar = 400 μm. Representative images from n = 5. (**a**) Undamaged media-only control. (**b, c**) Cells treated with (**b**) wild-type *S. aureus* strain 8325-4 or (**c**) the clinical strain USA300 JE2, showing integrity damage (white arrows). (**d–e**) Cells treated with (**d**) mutant *S. aureus* strains LES22 (SspA, -B, and -C deleted) or (**e**) USA300 DV8 (specific deletion of V8 protease) showing significantly less integrity damage. (**f**) Quantitation of integrity damage showing mean ± standard error of the mean for n = 3. ^∗^*P* < 0.05, ^∗∗∗^*P* < 0.001 versus parent strain.

The effects of secreted *S. aureus* proteases were examined in an in vitro monolayer damage assay, using HaCaT keratinocyte cells grown to confluency (Figure 1a–f). Application of bacterial supernatant from two different wild-type strains of *S. aureus* (8325-4 and USA300 JE2) produced visible areas of integrity damage (Figure 1b and c). No damage was observed in media-only controls (Figure 1a). Significantly less damage occurred when supernatants from mutant strains of *S. aureus* deficient in serine proteases SspA, SspB, and SspC (strain LES22 [Shaw et al., 2004]) or mutated to specifically delete SspA/V8 protease (strain USA300 DV8 [Fey et al., 2013]) were applied, compared with parent strain 8325-4 (Figure 1d–f). These data show a model of barrier integrity damage, induced by *S. aureus*-secreted proteases and requiring V8 protease.

### *S. aureus* SspA/V8 damages barrier integrity without causing cell death

**Figure 2:**
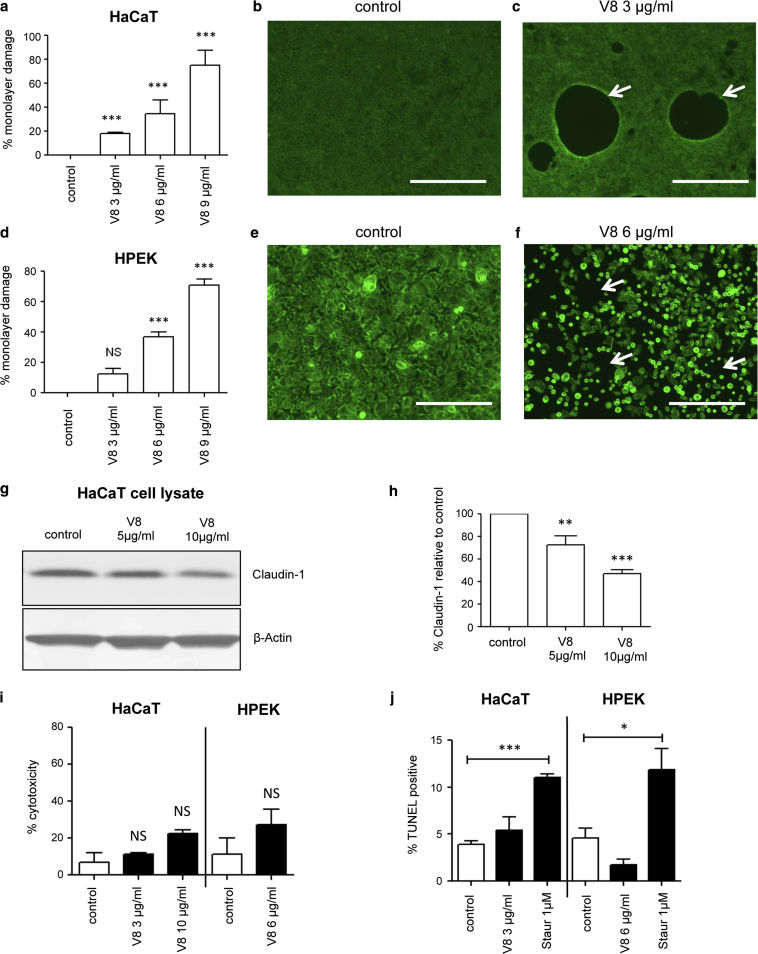
***Staphylococcus aureus* serine protease A (SspA/V8) damages keratinocyte barrier integrity without causing cell death.** HaCaT or HPEK cells were exposed to recombinant V8 protease for 24 hours. (**a–f**) Alexa488 Phalloidin staining and damage quantification. Mean ± standard error of the mean, n = 6. ^∗∗∗^*P* < 0.001 versus control. Scale bar = 400 μm. Arrows indicate damage. (**g, h**) Representative HaCaT lysate claudin-1 and β-actin Western blots and quantification. Mean ± standard error of the mean for n = 3. ^∗∗^*P* < 0.01, ^∗∗∗^*P* < 0.001 versus control. (**i, j**) Quantification of HaCaT (n = 3) or HPEK (n = 4) (**i**) lactate dehydrogenase release and (**j**) TUNEL staining, 1 μmol/L staurosporine used as TUNEL positive control. Mean ± SEM. ^∗^*P* < 0.05, ^∗∗∗^*P* < 0.001 versus control. HPEK, human primary epidermal keratinocyte; LDH, lactate dehydrogenase; M, mol/L; NS, not significant; Staur, staurosporine.

To establish whether V8 protease was sufficient to induce barrier integrity damage in the absence of other *S. aureus*-secreted factors, HaCaT cells were treated with recombinant V8 protease for 24 hours. Integrity damage was observed in a concentration-dependent manner (Figure 2a–c), with a phenotype similar to that observed using bacterial supernatant. Significant damage was observed at 3 μg/ml of V8, a concentration previously equated to protease levels found at *S. aureus*-colonized sites of AD patients (Miedzobrodzki et al., 2002). Significant and concentration-dependent V8 protease-mediated monolayer damage was also shown using human primary epidermal keratinocytes (HPEK) (Figure 2d–f), but showing a more diffuse integrity disruption phenotype (Figure 2d and f). These data show that V8 protease alone is sufficient to induce barrier integrity damage, both in primary and immortalized keratinocyte cultures.

*S. aureus* V8 protease has been proposed to cleave TJ proteins in damaging barrier integrity (Ohnemus et al., 2008). Consistent with this report, Western blotting of HaCaT keratinocyte lysates showed a significant reduction in claudin-1 after treatment with V8 protease in a dose-dependent manner (Figure 2g and h).

Measurements of cellular cytotoxicity (lactate dehydrogenase release, Figure 2i) or apoptosis (TUNEL staining, Figure 2j) showed no significant impact of V8 treatment in either HaCaT or HPEK cells. In contrast, treatment with staurosporine (as a positive control for apoptosis) induced a significant level of TUNEL staining in both cell types (Figure 2h and i) but did not induce the barrier integrity damage mediated by V8 (data not shown), showing that this phenotype was not dependent on cell death.

### Pretreatment with IL-1β induces protection of keratinocyte monolayers against *S. aureus* V8 protease-mediated damage

**Figure 3:**
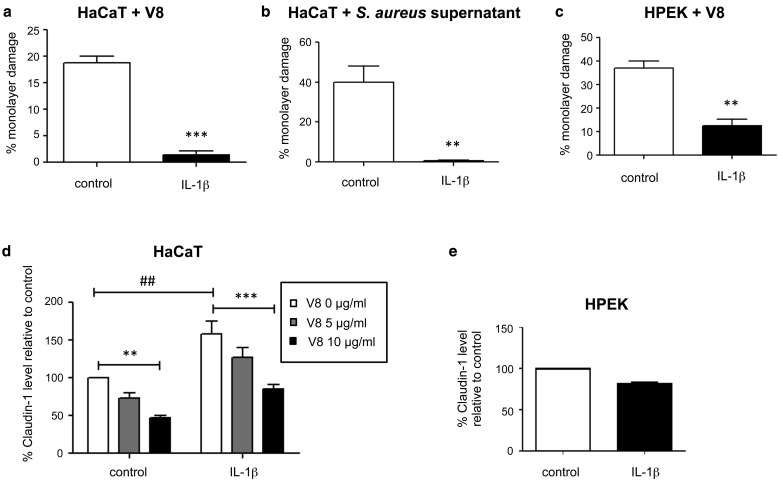
**Pretreatment with IL-1β protects keratinocytes from V8 protease-mediated damage.** (**a, b, d**) HaCaT or (**c, e**) HPEK cells were treated with IL-1β (100 ng/ml) or media only (control) for 24 hours, then exposed to (**a, c, d**) recombinant V8 protease or (**b**) *S. aureus* strain 8325-4 supernatant or (**e**) left untreated for 24 hours. (**a, b, c**) Integrity damage was quantified. Data show mean ± standard error of the mean for (**a**) n = 8 or (**b, c**) n = 3. ^∗∗^*P* < 0.01, ^∗∗∗^*P* < 0.001 versus control. (**d, e**) Western blot quantitation of claudin-1 normalized to control. Data show mean ± standard error of the mean for (**d**) n = 5 or (**e**) n = 3. ^∗∗^*P* < 0.01, ^∗∗∗^*P* < 0.001 for V8-treated versus untreated samples; ^##^*P* < 0.01 for IL-1β–treated versus untreated controls. HPEK, human primary epidermal keratinocyte.

IL-1β is a key proinflammatory cytokine proposed to modulate expression of TJ proteins and to have impaired production in AD (Abe et al., 2003, Jakob et al., 1995, Nomura et al., 2003). To establish whether IL-1β induced protection against *S. aureus* proteases, we treated HaCaT cells with IL-1β for 24 hours and exposed them to 3μg/ml of V8 protease (Figure 3a) or *S. aureus* supernatant (Figure 3b). IL-1β exposure induced significant protection against integrity damage from V8 protease (88% reduction, Figure 3a) and *S. aureus* supernatant (99% reduction, Figure 3b) in a concentration-dependent manner (see Supplementary Figure S1 online). This was also observed in primary cells exposed to 6μg/ml of V8 protease (Figure 3c).

Baseline levels of claudin-1 were significantly increased in HaCaT cells after IL-1β exposure (58% increase, Figure 3d), compatible with TJ-mediated enhanced protection against V8-induced integrity damage. However, IL-1β stimulation did not prevent V8 protease-mediated reduction of claudin-1 levels, with decreases of 53% and 46% (without and with IL-1β, respectively) induced by 10μg/ml of V8 protease (Figure 3d). Furthermore, in HPEK cells, claudin-1 was not significantly affected by pretreatment with IL-1β (Figure 3e), indicating dependence on alternative or additional IL-1β–induced mechanisms.

### IL-1β treatment of keratinocytes induces expression of a soluble protective factor

**Figure 4:**
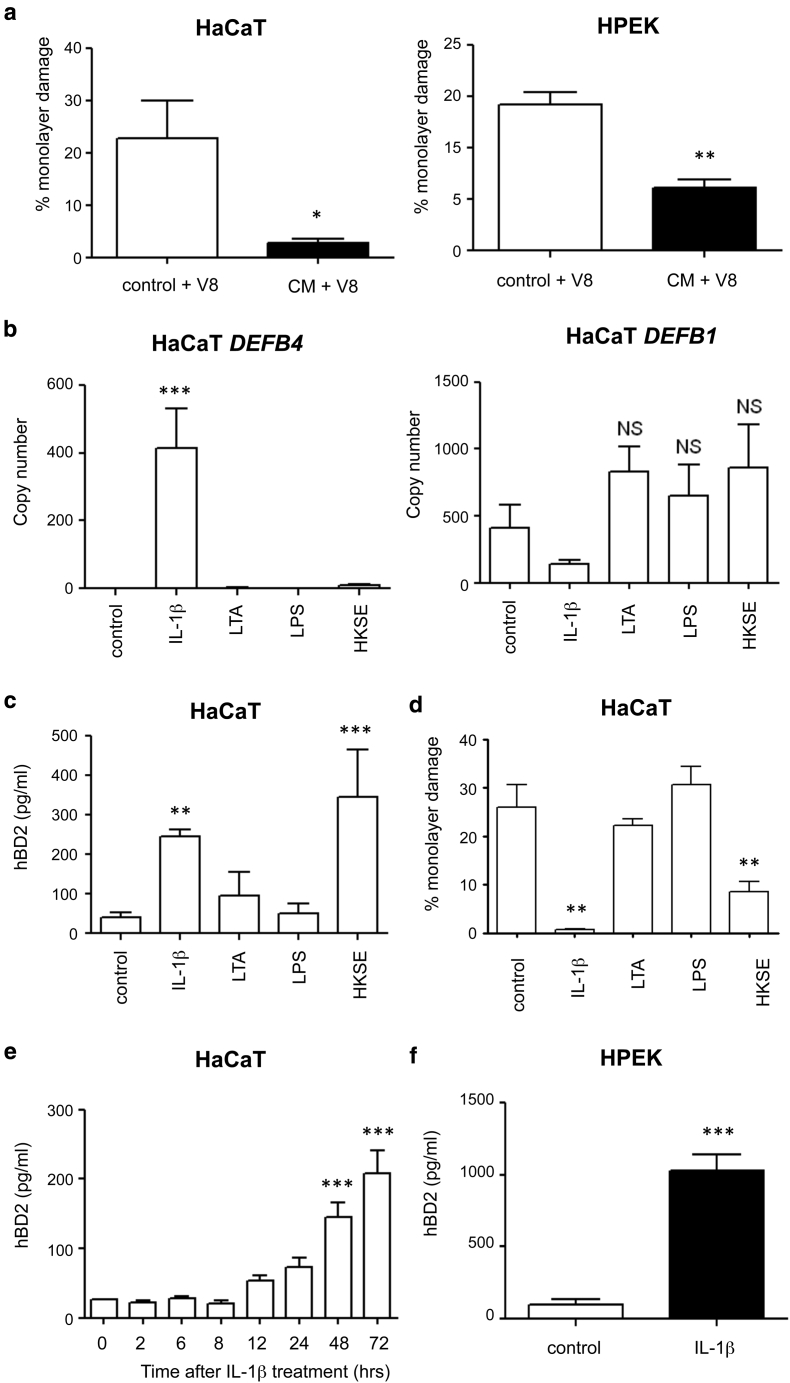
**IL-1β treatment induces the expression of hBD2 in keratinocytes, a soluble factor that protects against V8 protease-mediated damage.** (**a**) HaCaT (n = 9) or HPEK (n = 3) cells treated with conditioned media (transferred from control or IL-1β [100 ng/ml] pretreated cells) were exposed to recombinant V8 protease for 24 hours before quantification of damage. (**b–f**) HaCaT or HPEK cells treated for 24 hours or (**e**) over a time course with control (media only), IL-1β (100 ng/ml), LTA (10 μg/ml), LPS (5 μg/ml), or heat-killed *Staphylococcus epidermidis,* then assessed by (**b**) real-time reverse transcriptase PCR for expression of *DEFB4*, *DEFB1*, (**c, e, f**) ELISA for hBD2, and (**d**) quantification of V8-induced damage. Data show mean ± standard error of the mean. ^∗^*P* < 0.05, ^∗∗^*P* < 0.01, ^∗∗∗^*P* < 0.001 versus control. CM, conditioned media; HKSE, heat-killed *S. epidermidis*; HPEK, human primary epidermal keratinocyte; LPS, lipopolysaccharide; LTA, lipoteichoic acid; NS, not significant.

To determine the nature of IL-1β–induced protection against V8, media transfer studies were conducted between IL-1β–treated keratinocytes and naive cells before V8 exposure (Figure 4a). After 24 hours of exposure to IL-1β, media was discarded, cells were washed to remove residual IL-1β, and fresh media was added for 48 hours to generate conditioned media, which was transferred onto naive cells. Both naive HaCaT and HPEK cells exposed to conditioned media from IL-1β–treated cells (but not from control cells) were significantly protected against V8 protease (Figure 4a). These data show that IL-1β induces keratinocyte production of a secreted, transferrable protective factor.

IL-1β can stimulate expression of hBDs in keratinocytes, including hBD2 and hBD3 but not hBD1 (King et al., 2002). HaCaT cell expression of *DEFB1*, *DEFB4,* and *DEFB103* (encoding hBD1, hBD2, and hBD3, respectively) was therefore quantified by real-time reverse transcriptase PCR in cells exposed to IL-1β or to lipoteichoic acid (LTA), lipopolysaccharide (LPS), or heat-killed skin commensal *Staphylococcus epidermidis* (previously shown to suppress pathogens by stimulating HDP expression [Lai et al., 2010]). Transcription of *DEFB4* (hBD2) was not detected in unstimulated HaCaT cells, but it was significantly induced by IL-1β stimulation (Figure 4b) but not by the other stimuli. In contrast, *DEFB103* (hBD3) expression was not detected (data not shown) and, in agreement with published data, *DEFB1* (hBD1) expression was not significantly induced by any of these stimuli (Figure 4b). Keratinocyte production of hBD2 peptide was therefore examined by ELISA and found to be significantly induced by exposure to IL-1β (6-fold increase, to approximately 300 pg/ml), but not to LTA or LPS (Figure 4c). Heat-killed *S. epidermidis* also stimulated hBD2 release (9-fold increase), despite not affecting *DEFB4* transcription. Production of hBD2 correlated with protection against V8-mediated damage, with significant protection observed in cells treated with IL-1β or heat-killed *S. epidermidis* but not LTA or LPS (Figure 4d).

Given the correlation between hBD2 induction and protection, hBD2 was assessed as a candidate secreted, protective factor. A time course of IL-1β–mediated hBD2 induction in HaCaT cells showed a significant increase of secreted hBD2 at 48–72 hours after treatment with IL-1β (Figure 4e). These findings are compatible with the time point used in V8 damage and conditioned media transfer studies above (Figures 3a, 3b, and 4a). Significant induction of hBD2 was also observed in IL-1β–stimulated HPEK cells to approximately 1,000 pg/ml (9-fold increase, Figure 4f), correlating with significant protection from V8 damage (Figure 3c).

### Keratinocyte hBD2 production is sufficient to protect against V8 protease-mediated damage

**Figure 5:**
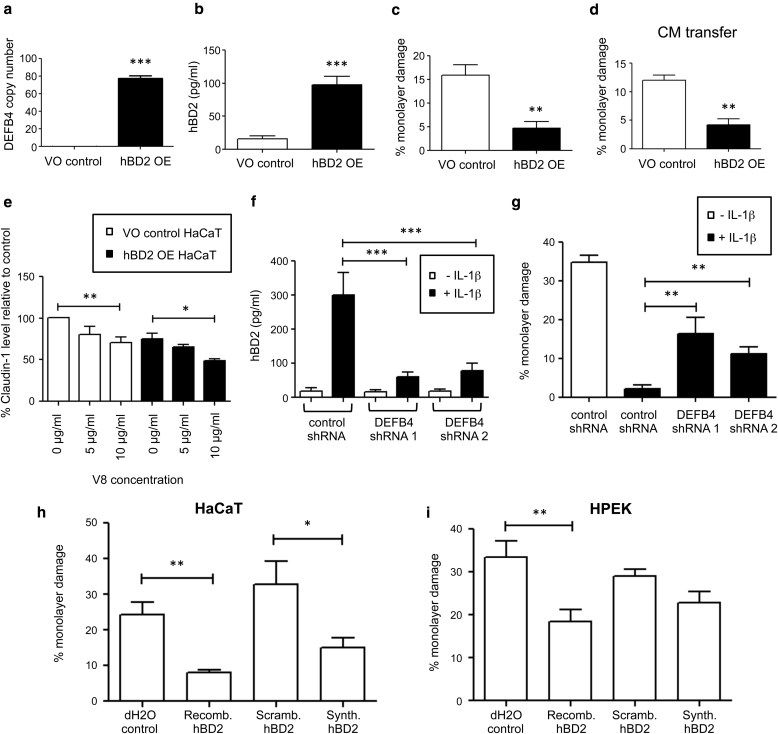
**hBD2 is sufficient and necessary to protect against V8 protease-mediated damage.** (**a–e**) hBD2 overexpressing (hBD2 OE) and vector only (VO control) HaCaT or naive HaCaT preincubated with CM or (**f, g**) *DEFB4* shRNA-knockdown and control shRNA HaCaT lines ± IL-1β prestimulation (100 ng/ml, 24 hours), assessed by (**a**) *DEFB4* quantitative real-time reverse transcriptase–PCR (n = 3), (**b, f**) hBD2 ELISA (n = 5–7), (**c, d, g**) V8-mediated damage quantification (n = 3–4), and (**e**) quantified Western blot ± V8 pre-exposure (3 μg/ml, 24 hours). (**h, i**) HaCaT or HPEK pretreated with distilled water, recombinant (3 μg/ml), scrambled (**h**: 3 μg/ml, **i**: 6 μg/ml), or synthetic hBD2 (**h**: 3 μg/ml, **i**: 6 μg/ml**)** 24 hours before V8 (**h**: 24 hours, 3 μg/ml; **i**: 48 hours, 6 μg/ml). n = 3–5. Mean ± standard error of the mean. ^∗^*P* < 0.05, ^∗∗^*P* < 0.01, ^∗∗∗^*P* < 0.001. CM, conditioned media; hBD, human β-defensin; HPEK, human primary epidermal keratinocyte; OE, overexpressing; Recomb., recombinant; Scramb., scrambled; shRNA, small hairpin RNA; Synth, synthetic; VO, vector only.

To determine whether hBD2 induction was causatively associated with protection against V8 protease, a *DEFB4* stably overexpressing HaCaT cell line (hBD2 OE) and corresponding vector-only control line (VO control) were constructed. Constitutive *DEFB4* transcription was shown in hBD2 OE cells without IL-1β stimulation (Figure 5a), and significant hBD2 peptide secretion was detected (at approximately 100 pg/ml, compared with <20pg/ml in VO control cells) (Figure 5b). Correspondingly, hBD2 OE cells were significantly protected against V8 protease compared with VO control cells (Figure 5c). In addition, transfer of conditioned media from hBD2 OE cells, but not from VO control cells, significantly protected naive HaCaT cells against V8 protease-mediated damage (Figure 5d). These data show that constitutive endogenous hBD2 expression, in the absence of IL-1β, is sufficient to replicate the IL-1β–mediated protective phenotype against V8-induced integrity damage.

Claudin-1 levels were also assessed in hBD2 OE (Figure 5e). V8 mediated significant reduction in claudin-1 levels in hBD2 OE and VO controls, but in contrast to IL-1β stimulation of HaCaT cells (Figure 3d), constitutive endogenous hBD2 expression did not increase claudin-1. These data confirm that hBD2-mediated protection is not a result of increased claudin-1 expression.

### Keratinocyte hBD2 production is required for IL-1β–mediated protection against V8 protease-induced damage

To establish whether hBD2 expression was required for IL-1β–mediated protection against V8 protease-induced keratinocyte integrity damage, two different stable HaCaT hBD2 small hairpin RNA (shRNA) knockdown keratinocyte cell lines (*DEFB4* shRNA 1 and 2) and a nontargeting control shRNA line were generated. hBD2 knockdown was verified by ELISA, shown by failure to induce hBD2 in response to IL-1β in either *DEFB4* shRNA line, with levels significantly (∼80%) lower than in the control (Figure 5f). Both *DEFB4* shRNA lines showed significant impairment of IL-1β–mediated protection against V8-induced monolayer damage compared with the nontargeting shRNA control line (Figure 5g). These data show that IL-1β–induced endogenous hBD2 expression is required for this protective phenotype.

### Addition of exogenous hBD2 protects against V8 protease-mediated damage

Having established that induction of endogenous hBD2 expression is protective against V8 protease-induced damage, the therapeutic potential of exogenously applied peptide was determined. Addition of either purified recombinant hBD2 or chemically synthesized hBD2 (but not a scrambled sequence version of the peptide or vehicle control) to unstimulated keratinocytes before V8 protease exposure significantly protected HaCaT cells (Figure 5h) and HPEK cells (Figure 5i) against damage. These data show that exogenously applied hBD2 can reconstitute the protective phenotype against V8 protease and raise the possibility of therapeutic applications.

## Discussion

The pathogenesis of AD and the role of innate host defense-mediated protection of epidermal integrity in AD remains incompletely understood. However, defects in epidermal barrier function in AD are highly associated with dry skin and hyperactive immune response to allergens (Cork et al., 2006, Sabin et al., 2012). In addition, recent clinical trials show that restoring and maintaining barrier integrity is critical in preventing AD onset in neonates (Horimukai et al., 2014, Simpson et al., 2014), confirming the fundamental importance of epidermal integrity. The capacity of proteases to induce barrier dysfunction is proposed as a key driving force in AD initiation and exacerbation (Takai and Ikeda, 2011). We therefore hypothesized that innate host defense against proteolytic virulence factors produced by the common AD skin pathogen *S. aureus* may be vital to maintaining barrier function.

To determine the impact of bacterial proteases on keratinocyte function, the effects of *S. aureus*-conditioned media (in the absence of live bacteria) were evaluated using immortalized and primary keratinocyte monolayer culture models. These studies generated an integrity damage model and established that the primary *S. aureus*-secreted factor was V8 protease (i.e., SspA). V8 exposure generated discrete “holes” in HaCaT monolayers without inducing cell death; experimental induction of apoptosis did not replicate this phenotype. In live cell imaging, rapid contraction from the center of such holes was observed (data not shown), with a concentrated band of phalloidin-positive signal seen around the “hole” edge (Figure 2c), suggestive of central foci of TJ failure and cell retraction from that point. In keeping with this, we show V8-cleavage of claudin-1 in this model. Primary keratinocytes were less sensitive to V8, perhaps because of higher baseline hBD2 production, requiring higher concentration of protease to reproducibly induce significant damage, but then undergoing a more catastrophic failure of monolayer integrity. These findings are compatible with previously shown V8-mediated damage to murine skin integrity in vivo (Hirasawa et al., 2010, Ohnemus et al., 2008), confirming the validity of this model and significance of this protease for keratinocyte barrier integrity.

A wide range of factors can promote keratinocyte defense against the deleterious effects of pathogens, including cytokines released by immune cells (e.g. IL-1β, IL-22), and bacterial factors such as LPS and LTA (Ganz and Lehrer, 1998, Liu et al., 2002, O'Neil et al., 1999). Evaluation of host and bacterial inflammatory agonists established that IL-1β, previously shown to modulate TJ protein expression and be diminished in AD (Abe et al., 2003, Jakob et al., 1995, Nomura et al., 2003), induced protection of keratinocytes against frank V8-mediated integrity damage. This effect was not observed after stimulation with LPS or LTA but was also generated by exposure to heat-killed *S. epidermidis*. Although IL-1β up-regulated claudin-1 expression in HaCaT cells, claudin-1 degradation was not inhibited, and protection was independent of claudin-1 levels in primary cells. These data indicated an alternative mechanism of protection, shown to be via secretion of an unidentified, transferrable innate host factor.

In contrast to patients with psoriasis, individuals with AD have impaired expression of HDPs, in particular hBD2, hBD3, and the cathelicidin LL-37 in inflamed lesions (Gambichler et al., 2008, Ong et al., 2002). Although several HDPs appear to have impaired expression in AD, hBD2 is reported to have the closest correlation with AD severity and skin infections (Clausen et al., 2013). In addition to direct antibacterial and antiviral properties (Gwyer Findlay et al., 2013, Lehrer et al., 1989, Mookherjee and Hancock, 2007), hBD2 also has modulatory effects on both the innate and adaptive immune system and promotes wound healing (Beaumont et al., 2014, Choi et al., 2012, Semple and Dorin, 2012, Yeung et al., 2011). Modulatory functions of HDP have key roles in the pathogenesis of psoriasis (Morizane and Gallo, 2012) and rosacea (Yamasaki et al., 2007) but are undescribed in AD. Unlike hBD1, which is constitutively expressed in skin, hBD2 and hBD3 expression are induced by proinflammatory cytokines, including IL-1β and bacterial products from commensals such as *S. epidermidis* (via a toll-like receptor 2–mediated pathway [Lai et al., 2010] as well as skin wounding [Ganz and Lehrer, 1998, Liu et al., 2002, O'Neil et al., 1999]). Induction of hBD2 in our keratinocyte models was found to correlate with the inducible protection phenotype in response to IL-1β and heat killed *S. epidermidis*. Genetic manipulation of hBD2 expression was then utilized to show that hBD2 production is the mechanism underpinning IL-1β–mediated protection against V8-induced barrier integrity damage. Keratinocyte production of hBD2 was required and sufficient (in the absence of IL-1β) for the protection phenotype, which was independent of effects on claudin-1 expression or degradation.

The cellular consequences of hBD2 production that lead to protection against V8 remain to be determined and are the subject of ongoing studies. Although hBD2, at concentrations of 7 μg/ml and above, can promote cell proliferation and tissue remodeling, and can induce matrix metalloproteinases and tissue inhibitors of metalloproteinase (Li et al., 2006), overexpression of hBD2 in our study did not affect cell proliferation (data not shown). β-Defensins are not characterized as exhibiting direct anti-protease activity; however, this has been reported for neutrophil α-defensins (Van Wetering et al., 1997) and other antimicrobial proteins including SLPI and elafin (Moreau et al., 2008, Williams et al., 2006). Furthermore, hBD2 and hBD3 contain an evolutionarily conserved “gamma-core” structural motif sufficient for antimicrobial activity and, interestingly, also for resistance to proteolytic degradation (Nigro et al., 2015). The significance of these structural features with respect to protection against V8 protease remains to be determined. Alternatively, hBD2 may induce keratinocyte production of anti-proteases, TJ proteins other than claudin-1, or alternative factors. Recent studies have shown that exposure to high concentrations of exogenously applied hBD3 and LL-37 can selectively increase keratinocyte expression of claudins and occludin to enhance TJ function (Akiyama et al., 2014, Kiatsurayanon et al., 2014). However, in keeping with our study, hBD2 was found not to enhance TJ function, and the consequence of these HDP-mediated effects on susceptibility to bacterial proteases was not determined. hBD3 was not expressed in our models, nor was LL-37 induced by IL-1β (data not shown) in our keratinocyte models. Despite an inability to enhance TJ function, we show that therapeutic application of exogenous recombinant or synthetic hBD2 (but not a scrambled sequence peptide control) could protect keratinocytes from V8-mediated barrier integrity damage without induction of endogenous peptide. This has clear implications for the development of therapies.

Because of the early onset and chronic relapsing nature of AD, safe, long-term therapeutics are required. However, the current principal treatment, topical corticosteroid, can have detrimental effects (e.g., perioral dermatitis and skin atrophy), and individuals becoming refractory to topical application require systemic treatment, with potentially severe adverse effects (Schakel et al., 2014). Development of interventions to restore barrier integrity was recently identified by researchers, clinicians, patients, and policymakers as a top-10 translational dermatology research priority in an e-Delphi exercise (Healy et al., 2015). Our data show that interventional approaches aimed at promoting endogenous hBD2 expression and/or the therapeutic application of hBD2 (or synthetic analogues) may have the potential to enhance/restore skin barrier integrity, protect against *S. aureus* V8 protease-mediated damage, and reduce reliance on corticosteroids.

## Material and Methods

Antibodies and real-time PCR reagents are listed in Supplementary Table S1 online.

### Bacterial strains

*S. aureus* strains 8325-4, LES22, USA300 JE2, and USA300 DV8 were a kind gift from Simon Foster and Lindsay Shaw (University of Sheffield), and *S. epidermidis* strain 9759 was from J. Ross Fitzgerald (University of Edinburgh). Bacteria were grown in 10 ml of tryptic soy broth (*S. aureus*) (with 5μg/ml erythromycin for LES22 and USA300 DV8) or luria broth (*S. epidermidis*) for 16 hours at 37 °C with shaking. Bacteria were harvested at an optical density (600 nm) of 1.4, pelleted for 10 minutes at 5,000*g*, resuspended in 1 ml of media, and either supernatant clarified with a 0.22-μm filter and concentrated in an RC1010 centrifugal evaporator (Jouan, Winchester, U.K.) (*S. aureus*) or bacteria heat killed at 70 °C for 30 minutes before use at an effective multiplicity of infection of 240:1 (*S. epidermidis*).

### Cell culture

Human keratinocyte line HaCaT (CLS GmbH, Eppelheim, Germany) was maintained in DMEM (DMEM/F-12, Gibco, Loughborough, UK) with 10% volume-to-volume fetal bovine serum and 1% penicillin 100IU/ml–streptomycin 10μg/ml–2mmol/L l-glutamine (GE Healthcare, Buckinghamshire, UK). HPEKp0.5 cells (CELLnTEC, Bern, Switzerland) were maintained in CnT57 media (CELLnTEC) and replaced with CnT02 media (CELLnTEC) at 100% confluency. All cells were cultured at 37 °C, 95% relative humidity, and 5% CO_2_.

To create hBD2 OE and VO control HaCaT lines, TrueORF Gold pCMV6-DEFB4 gene expression clone or pCMV6 vector-only backbone (OriGene Technologies, Rockville, MD) were transfected with Lipofectamine 2000 (Life Technologies, Renfrew, UK), followed by selection with 500 μg/ml of G418 (Life Technologies). Individual hBD2 shRNA knockdown lines were made by stable transfection of HaCaT cells with pLKO.1 lentiviral shRNA target gene set targeting *DEFB4A* (GE Healthcare, catalogue no. RHS4533) or pLKO.1 vector containing a scrambled nontargeting shRNA hairpin (Addgene plasmid 1864, Cambridge, MA [Sarbassov et al., 2005]), followed by 5μg/ml puromycin selection.

### Keratinocyte monolayer damage assay

HaCaT or HPEK cells were seeded at 3 × 10^5^ cells/well in 12-well plate at 37 °C until monolayer formation (48–72 hours). Media was removed, cells washed twice with phosphate buffered saline, and serum-free media added. As required, monolayers were stimulated with 100ng/ml IL-1β (Peprotech, Rocky Hill, NJ), 10μg/ml of LTA (InvivoGen, San Diego, CA), 5μg/ml of LPS (InvivoGen), or heat-killed *S. epidermidis* or pretreated with 3 μg/ml of recombinant hBD2 peptide (ProSpec, East Brunswick, NJ), custom synthetic hBD2 (GIGDPVTCLKSGAICHPVFCPRRYKQIGTCGLPGTKCCKKP) or scrambled (IGKILKHVGLSGYCKGDCTRGPCGPFVITCCQCRKPPPAKT) peptides (Almac, East Lothian, UK) for 24 hours. Subsequently, recombinant *S. aureus* V8 protease (Worthington, Lakewood, NJ) was added as indicated. Cell supernatant was collected for ELISA, and protein lysate or RNA was extracted from treated cells.

Damage was assessed from images acquired using an EVOS *fl* digital inverted microscope (ThermoFisher Scientific, Paisley, UK). Representative images (>3 per condition) were analyzed using ImageJ software (National Institutes of Health, Bethesda, MD), quantifying breaks in the keratinocyte monolayer, with damage expressed as a percentage loss of monolayer integrity. For phalloidin staining, treated keratinocyte monolayers were fixed in 4% PFA, washed in phosphate buffered saline, and incubated with Alexa488 phalloidin (Life Technologies) per the manufacturer’s instructions.

### SDS-PAGE and Western blot

Cell lysates were prepared using M-PER Mammalian Protein Extraction Reagent (Life Technologies), equalized, and boiled at 96 °C for 5 minutes in loading/reducing buffer. Next, 20-μg samples were electrophoresed on 4–12% SDS-PAGE Novex gels (Life Technologies), transferred to 0.2-μmol/L nitrocellulose membrane (Life Technologies) using Towbin buffer, blocked with 5% milk in Tris-buffered saline + 0.1% Tween-20, (Sigma Aldrich, Dorset, UK) and incubated with antibodies as detailed. Western blots were visualized and quantitated using the LI-COR Biosciences (Lincoln, NE) Odyssey system.

### Real-time PCR

RNA was extracted from keratinocytes using RNeasy mini kit (Qiagen, Manchester, UK) and reverse transcribed to cDNA using the TaqMan RT-PCR kit (Life Technologies). StepOne Real Time PCR machine (ThermoFisher Scientific) and 48-well MicroAmp optical plates (Life Technologies, Renfrew, UK) were used for amplification reactions. Copy number was analyzed according to McNeilly et al. (2008). Standard curves were performed using plasmids for *DEFB1*, *DEFB4* (Origene #SC116851, RC219487), and *DEFB103* (generated by J. Dorin).

### ELISA

hBD2 ELISA development kit (Peprotech) was used to determine hBD2 peptide levels in culture media per the manufacturer’s protocol. Peptide levels were determined in neat supernatant in triplicate.

### Cytotoxicity and apoptosis measurements

Lactate dehydrogenase release was assayed using the lactate dehydrogenase cytotoxicity detection kit (Abcam, Cambridge, UK). Absorbance was measured at 450 nm, with a reference wavelength of 650 nm. Cytotoxicity was calculated using the formula: cytotoxicity (%) = (test sample – low control [untreated cells])/(high control [lysed cells] – low control) × 100. Apoptosis was assessed by TUNEL assay using the In Situ Cell Death Detection Kit (Roche, Basel, Switzerland). Cells were treated with the conditions indicated and compared with treatment with staurosporine at 1 μmol/L for 3 hours as a positive control.

### Statistical analysis

Statistical analysis was performed using the GraphPad PRISM5 statistical package (GraphPad software, La Jolla, CA) by Student *t* test (Figures 1f and 2i (HPEK), 4a and f, and 5a–d and g–i), one-way analysis of variance with Dunnett multiple comparison posttest (Figures 2a, d, h, i [HaCaT] and j; 3 a–c and e; and 4 b–e) or two-way analysis of variance with Bonferroni posttest (Figures 3d and 5e and f). *P*-values below 0.05 were considered significant.

## Conflict of Interest

The authors state no conflict of interest.
