# Supplementary material for: IL-1β–Induced Protection of Keratinocytes against Staphylococcus aureus-Secreted Proteases Is Mediated by Human β-Defensin 2
Source: J Invest Dermatol. 2017 Jan;137(1):95–105. doi: 10.1016/j.jid.2016.08.025 (PMC5176011; doi:10.1016/j.jid.2016.08.025)
Supplement: Supplementary Figure S1 and Supplementary Table S1 [file mmc1.pdf]

## Supplementary Figure S1

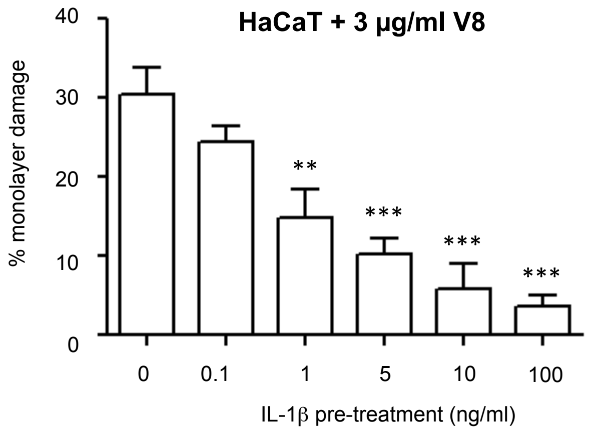


**Supplementary Figure S1**. **Dose response curve of IL-1β-mediated keratinocyte protection.** HaCaT cells were pre-treated with IL-1β at the concentrations shown for 24 hours, then exposed to 3 μg/ml recombinant V8 protease for 24 hours. Integrity damage was quantified, data showing mean +/- SEM for n = 3, ** p<0.01, *** p<0.001 versus untreated media-only control.

## Supplementary Table S1

| | | |
|---|---|---|
| **Antibodies** | | |
| Claudin-1 | Rabbit anti-human | Life Technologies |
| Beta-actin | Mouse anti-human | Sigma Aldrich |
| IRDye 800CW secondary antibody | Goat anti-rabbit | LI-COR Biosciences |
| IRDye 688CW secondary antibody | Goat anti-mouse | LI-COR Biosciences |
| | | |
| **Real Time PCR** | **TaqMan assay ID no.** | |
| DEFB1 | Hs00608345_m1 | Life Technologies |
| DEFB4 | Hs00823638_m1 | Life Technologies |
| DEFB103 | Hs04194486_g1 | Life Technologies |
